# CURRENT STATE OF SCIENCE ON DRUG THERAPIES FOR OSTEOPOROTIC FRACTURE PREVENTION

**DOI:** 10.1093/geroni/igz038.2744

**Published:** 2019-11-08

**Authors:** Sundeep Khosla

**Affiliations:** Mayo Clinic, Rochester, Minnesota, United States

## Abstract

During the workshop, experts discussed the state of the science. The U.S. Food and Drug Administration (FDA) has approved several medications to prevent osteoporotic fractures. These include bisphosphonates (BPs), denosumab, teriparatide, estrogens, and selective estrogen-receptor modulators. BPs are a first line of pharmacologic treatment for most women and men who have osteoporosis. Despite the efficacy of short-term use of BPs and other osteoporosis medications, treatment rates are low and appear to be decreasing. This talk would describe the problem/epidemiology of fracture prevention in the United States; the importance of drug therapy and their effectiveness; the limited evidence regarding long-term use of drug therapies; the questions surrounding the application of drug holidays; and the complex physician and patient factors that impact the use of and adherence to osteoporotic drugs.

